# Supplementary material for: Multiproxy stable isotope analysis provides insights into diet, animal management, and residential mobility in Old Bara, a metropolitan suburb of the Oyo Empire, West Africa
Source: PLoS One. 2026 Apr 6;21(4):e0345981. doi: 10.1371/journal.pone.0345981 (PMC13052869; doi:10.1371/journal.pone.0345981)
Supplement: S1 Data — (DOCX) [file pone.0345981.s001.docx]

Fig 1: Variation in Carbon (*δ*^13^C) isotope values within the tooth of three caprines from Old Bara

Fig 2: Variation in nitrogen (*δ*^15^N) isotope values within the tooth of three caprines from Old Bara

**Zooarchaeology by Mass Spectrometry (ZooMS) Results**

Twenty-one collagen samples from faunal remains at Old Bara were analyzed using peptide mass fingerprinting to clarify some individuals identified with low confidence via morphology and to push the identification of Caprinae to the genus level. Of these, 19 could be identified to some taxonomic level or at least rule out some species (Table 1). In general, the ZooMS identifications were consistent with those based on morphology. Members of the family Bovidae are difficult to identify to the species level, due to similarities in their collagen type I sequences, although some genera have diagnostic markers (1). Specially, goat (*Capra* sp.) can be easily differentiated by the presence of m/z 3093 at COL1ɑ2 757 – 789 (G Marker) (2, 3) (Figure 3). In contrast, sheep (*Ovis* sp.) share peptide markers with other African bovids, especially members of the subfamily Alcelaphinae, and may be difficult to confidently identify to species if some peptide markers are absent. In these cases, morphology is essential for narrowing down potential identifications. In this study, five of the seven successful equid identifications were confirmed with ZooMS, with one sample identified as an Artiodactyl. Of the seven successfully identified caprine samples, three were identified as goats, three were consistent with sheep, and one was identified as a non-caprine bovid. The sheep and goat are morphologically consistent with West African Dwarf (WAD) sheep and goat. Additional morphological assessment of the non-caprine bovid identified by ZooMS shows that these femoral shaft fragments are most consistent with common duiker (*Sylvicapra grimmia*). Due to a lack of available collagen, the equids could not be identified to the species level (i.e., horse or donkey) using a chymotrypsin digestion (4). The averaged MALDI-TOF mass spectra for all samples have been uploaded to the data repository Borealis (borealisdata.ca) under DOI: https://doi.org/10.5683/SP3/WKDYRV.


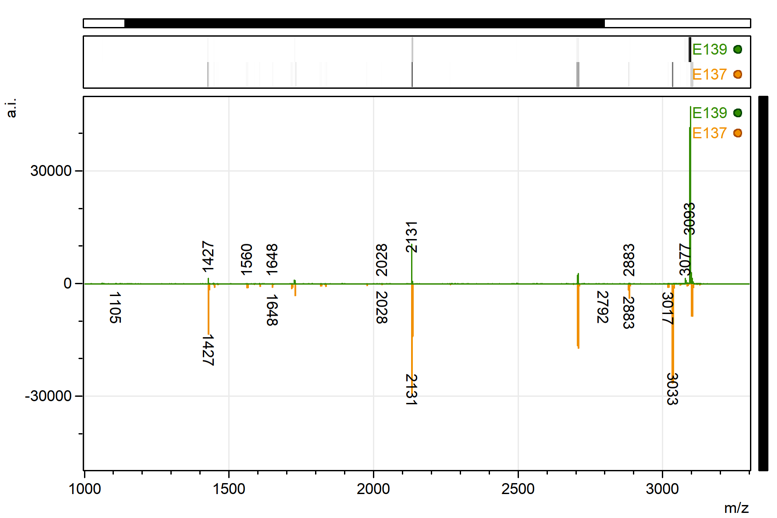


Figure 3: Spectra consistent with goat (top) and sheep (bottom), although ZooMS cannot definitively rule out other potential African bovids from the latter species.

Table 1: Results of ZooMS analysis on the Old Bara samples

| **ADαPT Code** | **TEAL ID** | **Morphological ID** | **ZooMS ID** | **Comment** |
| --- | --- | --- | --- | --- |
| E117 | 27102 | Oribi/Carnivora? | Bovidae | Excluded from this study |
| E118 | 27104 | Harnessed bushbuck? | Tragelaphini |  |
| E119 | 27105 | Harnessed bushbuck | Tragelaphini |  |
| E120 | 27224 | Cattle? | Bos sp. |  |
| E121 | 27225 | Cattle? | Possible *Equus* sp. | Confirmed as *Equus* sp. |
| E122 | 27226 | Cattle? | Possible Alcelaphus | Confirmed as *Alcelaphus buselaphus* |
| E123 | 27228 | Equid | No ID |  |
| E124 | 27229 | Equid | *Equus* sp. |  |
| E125 | 27232 | Equid | *Equus* sp. |  |
| E126 | 27233 | Equid | *Equus* sp. |  |
| E127 | 27237 | Equid | Artiodactyla | Excluded from this study |
| E129 | 27241 | Equid | *Equus* sp. |  |
| E130 | 27243 | Equid | *Equus* sp. |  |
| E132 | 27255 | Caprine | *Capra* sp*.* |  |
| E133 | 27256 | Caprine | *Capra* sp*.* |  |
| E134 | 27258 | Sheep | *Ovis* sp. |  |
| E135 | 27259 | Caprine | Non-Caprine bovid; consistent with Tragelaphini, *Sylvicapra*, or *Philantomba* | Confirmed as *Sylvicapra grimmia* |
| E136 | 27260 | Caprine | *Ovis* sp. |  |
| E137 | 27261 | Sheep | *Ovis* sp. |  |
| E138 | 27262 | Caprine | No ID |  |
| E139 | 27263 | Caprine | *Capra* sp. |  |

References

1. Janzen A, Richter KK, Mwebi O, Brown S, Onduso V, Gatwiri F, et al. Distinguishing African bovids using Zooarchaeology by Mass Spectrometry (ZooMS): New peptide markers and insights into Iron Age economies in Zambia. PLOS ONE. 2021;16(5):e0251061.

2. Buckley M, Collins M, Thomas-Oates J, Wilson JC. Species identification by analysis of bone collagen using matrix-assisted laser desorption/ionisation time-of-flight mass spectrometry. Rapid Commun Mass Spectrom. 2009;23(23):3843-54.

3. Buckley M, Whitcher Kansa S, Howard S, Campbell S, Thomas-Oates J, Collins M. Distinguishing between archaeological sheep and goat bones using a single collagen peptide. Journal of Archaeological Science. 2010;37(1):13-20.

4. Paladugu R, Richter KK, Valente MJ, Gabriel S, Detry C, Warinner C, Dias CB. Your horse is a donkey! Identifying domesticated equids from Western Iberia using collagen fingerprinting. Journal of Archaeological Science. 2023;149:105696.
